# Gene-based genome-wide association study identified 19p13.3 for lean body mass

**DOI:** 10.1038/srep45025

**Published:** 2017-03-21

**Authors:** Shu Ran, Lei Zhang, Lu Liu, An-Ping Feng, Yu-Fang Pei, Lei Zhang, Ying-Ying Han, Yong Lin, Xiao Li, Wei-Wen Kong, Xin-Yi You, Wen Zhao, Qing Tian, Hui Shen, Yong-Hong Zhang, Hong-Wen Deng

**Affiliations:** 1 Center of System Biomedical Sciences, University of Shanghai for Science and Technology, Shanghai, PR China; 2 Center for Genetic Epidemiology and Genomics, School of Public Health, Soochow University, Jiangsu, PR China; 3 Jiangsu Key Laboratory of Preventive and Translational Medicine for Geriatric Diseases, Soochow University, Jiangsu, PR China; 4 Department of Epidemiology and Statistics, School of Public Health, Soochow University, Jiangsu, PR China; 5 Department of Biostatistics, Tulane University, New Orleans, Louisiana, USA

## Abstract

Lean body mass (LBM) is a complex trait for human health. To identify genomic loci underlying LBM, we performed a gene-based genome-wide association study of lean mass index (LMI) in 1000 unrelated Caucasian subjects, and replicated in 2283 unrelated Caucasians subjects. Gene-based association analyses highlighted the significant associations of three genes *UQCR, TCF3* and *MBD3* in one single locus 19p13.3 (discovery *p* = 6.10 × 10^−5^, 1.65 × 10^−4^ and 1.10 × 10^−4^; replication *p* = 2.21 × 10^−3^, 1.84 × 10^−3^ and 6.95 × 10^−3^; combined *p* = 2.26 × 10^−6^, 4.86 × 10^−6^ and 1.15 × 10^−5^, respectively). These results, together with the known functional relevance of the three genes to LMI, suggested that the 19p13.3 region containing *UQCR, TCF3* and *MBD3* genes was a novel locus underlying lean mass variation.

The muscular tissue, as characterized by lean body mass (LBM), is related to human health. Low LBM may be related to a series of health problems, such as sarcopenia, obesity, and increased mortality^1^,^2^. LBM is under genetic control, with heritability over 50%^3^,^4^. Previous GWA studies have found novel single nucleotide polymorphisms (SNPs) and genes associated with LBM^5^,^6^,^7^. However, the vast majority of LBM candidate genes remain to be revealed. LBM can be measured accurately by dual energy X-ray absorptiometry (DXA). Body lean mass index (LMI) is frequently used to predict sarcopenia^8^,^9^.

Genomic regions may present allelic heterogeneity to the phenotype, i.e., multiple variants in a region affect the phenotype jointly. In the presence of allelic heterogeneity, gene-based association test can improve statistical power and robustness of genetic association analysis by integrating multiple SNP signals into a single statistic^10^,^11^. A variety of statistical methods were developed for gene-based test, such as Versatile Gene-based Association Study (VEGAS)^12^. VEGAS combines *p*-values of multiple SNPs within a gene region into a gene-based score while accounting for linkage disequilibrium (LD) by simulating genotype data from a multivariate normal distribution in permutation-based test. VEGAS is computationally efficient because the number of permutation simulations is adaptive. Its performance is similar to other statistical methods^12^, but is superior to the others in certain conditions due to its usage of large population reference panels when performing permutation. Therefore, it uses summary statistics only instead of raw genotype and phenotype data, making it suitable for the summary results from large-scale meta-analyses.

In this study, we reported a gene-based GWAS for LMI to identify genetic loci underlying variation of LBM. The discovery sample included 1000 unrelated Caucasian subjects genotyped with the Affymetrix 500 k genotyping array. The replication sample included 2283 unrelated subjects of Caucasian subjects, genotyped with the Affymetrix SNP6.0 genotyping array. Genotypes in both samples were imputed with the 1000 genomes project sequencing reference panel.

## Materials and Methods

### Ethics Statement

Study participants were recruited from the cities of Omaha and Kansas city and their neighboring areas. The study was approved by institutional review boards of the Creighton University and the University of Missouri-Kansas city. All participants provided written informed consent documents before entering the study. The methods carried out in accordance with the approved study protocol.

### Subjects

#### Discovery sample

The discovery sample consisted of 1,000 unrelated Caucasian subjects of European ancestry, of whom 501 were males and 499 were females. The sample was randomly selected from a large-scale cohort containing over 6000 subjects. The inclusion and exclusion criteria for cases were described in our previous publications^13^.

#### Replication sample

The replication sample consisted of 2283 unrelated subjects of European ancestry. There were 556 male subjects and 1727 female subjects. All subjects were healthy individuals recruited from the Midwestern United States. There was no overlap between the subjects of the discovery and the replication cohorts.

### Phenotyping

All subjects completed a structured questionnaire including lifestyle, medical history, family information, anthropometric variables, etc. Lean body mass and fat body mass (FBM) were measured with a Hologic QDR 4500 W DXA scanner (Hologic Inc., Bedford, MA, USA). Weight was measured in light clothing, on a calibrated balance beam scale. Height was obtained using a calibrated stadiometer. Lean mass index (LMI, kg/m^2^) was calculated as the ratio of lean mass to square of height^14^.

### Genotyping and quality control

Genomic DNA was extracted from peripheral blood leukocytes using a commercial isolation kit (Gentra Systems, Minneapolis, MN, USA). Genotyping was performed as described in our previous publication^15^. Briefly, the discovery cohort was genotyped with the Affymetrix Mapping 250 K Nsp and Affymetrix Mapping 250 K Sty arrays at the Vanderbilt Microarray Shared Resource at Vanderbilt University Medical Center (Nashville, TN, USA) using the standard protocol recommended by the manufacturer (Affymetrix, Inc., Santa Clara, CA, USA). The Caucasian replication cohort was genotyped using the Affymetrix SNP 6.0 arrays by the standard protocol of the manufacturer.

We followed strict quality control (QC) procedure. Samples that had a minimum call rate of 95% were included. We discarded SNPs that deviated from Hardy-Weinberg equilibrium (*p* < 0.0001) and those containing a minor allele frequency (MAF) less than 0.01. After QC, 379,319 SNPs remained in the discovery sample.

### Genotype imputation

Genotype imputation was applied to both the discovery and replication samples, with the 1000 Genomes projects sequence variants as reference panel (as of August 2010). Reference sample included 283 individuals of European ancestry.

The details of genotype imputation process had been described earlier^16^. Briefly, strand orientations between reference panel and test sample were checked before imputation, and inconsistencies were resolved by changing the test sample to reverse strand or removing the SNP from the test sample. Imputation was performed with MINIMAC^17^. Quality control was applied to impute SNPs with the following criteria: imputation r^2^ > 0.5 and MAF > 0.01. SNPs failing the QC criteria were excluded from subsequent association analyses.

### SNP-based association analyses

We used VEGAS for gene based association analyses, which takes individual SNP association *p*-values as input. Therefore, we performed SNP-based association analyses first to generate association signals. We used the principal component based approach for the correction of population stratification problem^18^. In both samples, covariates including gender, age, age^2^, fat body mass (FBM) and the first five principal components^19^,^20^ derived from genome-wide genotype data were screened for significance with the step-wise linear regression model implemented in the R function stepAIC. Raw BMI values were adjusted by significant covariates (age, gender and FBM), and the residuals were normalized by inverse quantiles of standard normal distribution.

Genetic associations were examined between genotyped and/or imputed SNPs and normalized phenotypes under an additive mode of inheritance with MACH2QTL^21^,^22^, which fitted phenotype by allele dosage with a linear regression model.

Linkage disequilibrium (LD) measure r^2^ was calculated with Haploview^23^. To examine potential confounding effect caused by population stratification, we estimated the genomic control inflation factor (*λ*)^24^.

### Gene-based test using VEGAS

Gene-based association test was examined to identify genes that were associated with the phenotype. We used the VEGAS approach for such analysis, which requires LD structure information and individual SNP *p*-values as input^12^. The SNP inclusion criteria was the same as that for individual SNP tests. Specifically, we required that SNP imputation accuracy r^2^ > 0.5 and MAF > 0.01.

### Meta-analysis

Significant genes identified in the discovery sample were further replicated in the replication sample. The two gene-based association signals were then jointly analyzed with the Fisher’s method. Specifically, the Fisher’s statistic was calculated as


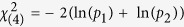


where *p*_1_ and *p*_2_ were the two gene-level p-values. Under the null hypothesis of no association, this statistic approximately follows the chi-square distribution with 4 degrees of freedom. Note that the Fisher’s method is always valid regardless of whether the directions of two effect sizes are consistent.

### Gene-level effect direction evaluation

To evaluate the consistency of two gene-level effect directions, we studied the effects of all individual SNPs within the gene and proposed an overall gene-level effect direction measure. We then compared the directions of the two measures. To accomplish this, we set all the SNP effects in the discovery sample to be positive so that a gene-level z-score is estimated by 
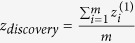
, where 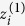 is the positive z-score of the *i*th SNP in the discovery sample. If the reported SNP z-score is negative, then the reference allele and the alternative allele will exchange so that the z-score changes to be positive. For example, if the reference allele and the alternative allele are *A* and *G*, and the reported z-score of allele *A* is −1.0, then the allele *G*, whose z-score is 1.0, will change to the reference allele.

Once all reference alleles are determined by referring to the discovery sample, we will calculate a gene-level z-score for the replication sample as 
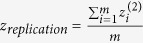
, where 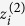 is the z-score of the reference allele at the *i*th SNP. If the *z*_*replication*_ is positive, then it has the same direction as *z*_*discovery*_, and we declare that the two genes are consistent in effect direction; otherwise, they are opposite in effect direction.

## Results

The basic characteristics of the subjects used in both discovery and replication samples are summarized in Table 1.

### Discovery sample

**Figure 1:**
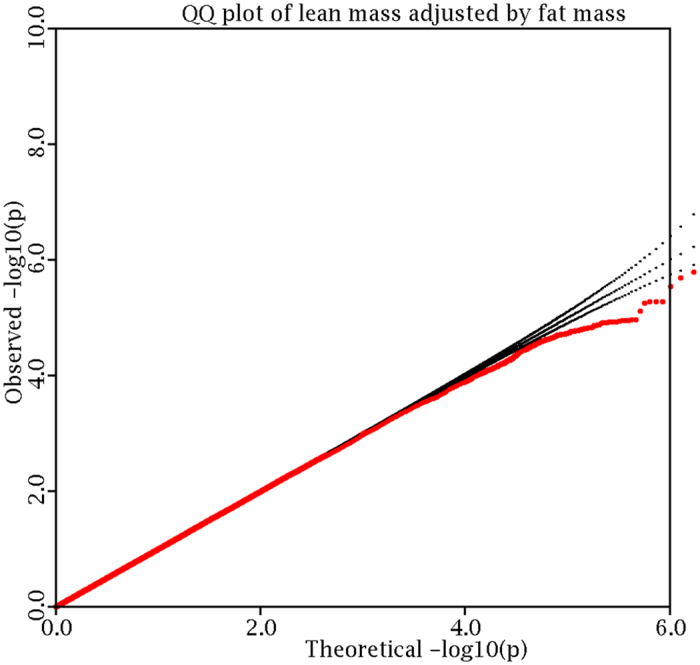
Logarithmic quantile–quantile (QQ) plot of individual SNP-based association.

**Figure 2:**
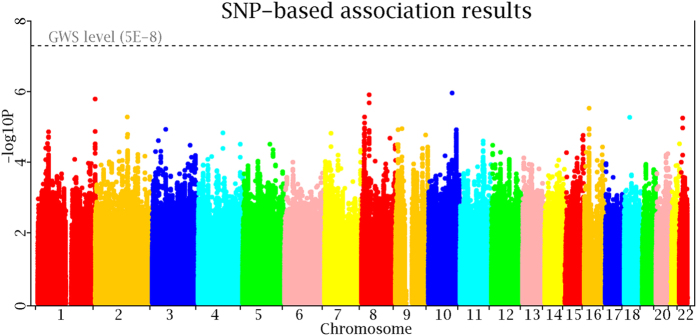
Manhattan plot of individual SNP-based association tests.

Using the genotyped and imputed genotypes in our sample of 1000 Caucasian subjects with GWAS data, we tested SNPs for association with LMI. Overall genomic control inflation factor was 1.05. To correct for potential population stratification, we adjusted individual p-values by the inflation factor. The logarithmic quantile–quantile (QQ) plot of SNP-based association results is displayed in Fig. 1. No evidence of population stratification is observed after adjustment by the genomic control approach. Manhattan plot of association results across the genome is displayed in Fig. 2. There is no genome-wide significant association signal.

**Figure 3:**
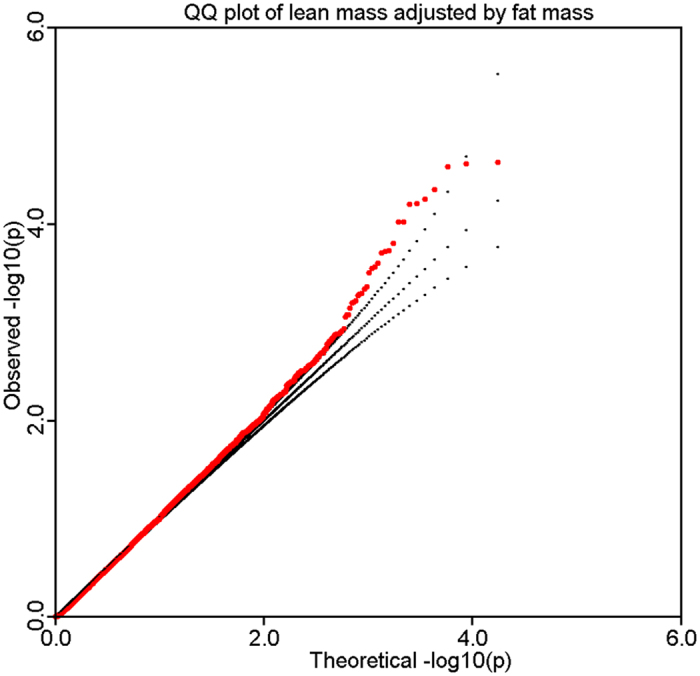
QQ plot of gene-based association tests.

**Figure 4:**
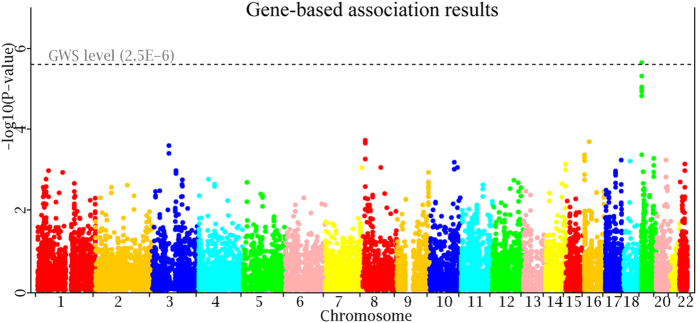
Manhattan plot of gene-based association tests.

Through individual SNP-based tests, we performed gene-based tests using VEGAS software. A total of 17,511 genes are included in the gene-based association test in the discovery sample. The Bonferroni correction is used to declare the genome-wide significance level (GWS, 0.05/17,511 = 2.86 × 10^−6^). The QQ plot of gene-based association results is displayed in Fig. 3. A marked deviation is observed in the tail of the distribution, implying true association signals. The Manhattan plot of gene-based association results is displayed in Fig. 4.

None of the genes is significant at the GWS level. At a less stringent borderline level 2 × 10^−4^, there are 11 genes that are significant. The most significant gene is *GATA4* at 8p23.1 (p = 2.40 × 10^−5^). These 11 genes are located into 6 distinct genomic regions.

### Replication sample

The 11 suggestively associated genes in the discovery sample are subjected to replication in the replication sample. SNP-based and gene-based association analyses in the replication sample are same to those in the discovery sample. Only three genes from the same locus 19p13.3 (*UQCR* p = 2.21 × 10^−3^, *TCF3* p = 1.84 × 10^−3^, *MBD3* p = 6.95 × 10^−3^), while the other 8 genes are not significant.

In the meta-analysis of both the discovery and the replication samples by the Fisher’s method, the signal of *UQCR* achieves the GWS level (discovery p = 6.10 × 10^−5^, replication p = 2.21 × 10^−3^, combined p = 2.26 × 10^−6^), while the signals of *TCF3* and *MBD3* are close to the GWS level (*TCF3* combined p = 4.86 × 10^−6^, *MBD3* combined p = 1.15 × 10^−5^).

Combining the evidence from both the discovery and the replication samples, three genes (*UQCR, TCF3* and *MBD3*) from one single locus 19p13.3 are convincingly associated with LMI after adjustment by fat mass. The main results are listed in Table 2.

**Figure 5:**
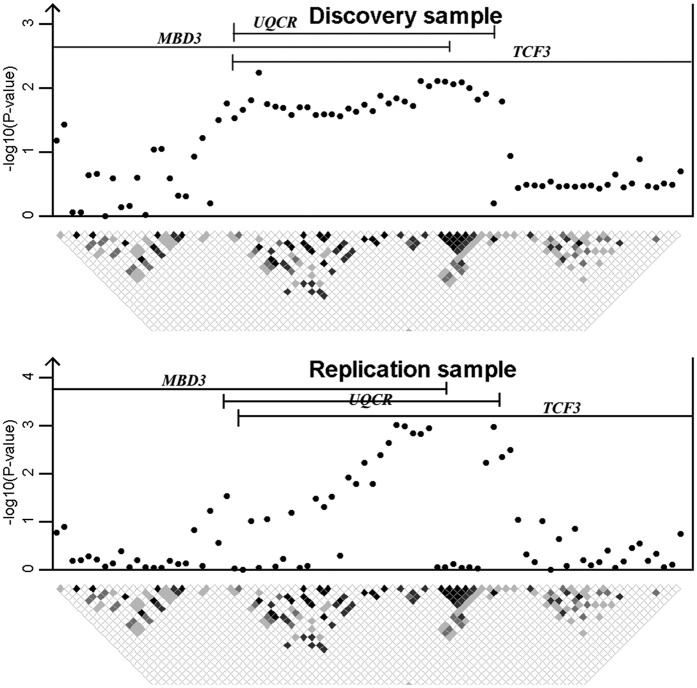
Regional plot of SNP associations around 19p13.3.

These three genes are apart 170 kb at most. Their tested regions overlap with each other. SNPs in this locus are divided into several haplotype blocks. The regional LD structure and association signals are displayed in Fig. 5.

### Gene-level effect direction evaluation

We evaluated the consistency of gene-level effect directions between the discovery and the replication samples. We proposed a gene-level effect direction measure in each study and then compared the two measures. The results are listed in Table 2. The z-scores in the discovery sample are always positive by definition. It is clear from the table that the z-scores in the replication sample are also positive in all cases, implying that the two studies are consistent in effect direction for all the three evaluated genes.

## Discussion

In this study, we have performed a genome-wide association study in 1000 unrelated Caucasian subjects and replicated in 2283 unrelated Caucasian subjects. Combining evidence from both the discovery sample and the replication sample, we have identified three genes *UQCR, TCF3* and *MBD3* located at 19p13.3 that were associated with LMI.

Gene density at chromosome 19 is the highest in all the human chromosomes, more than double of the genome-wide average level. The densely located gene families of repetitive DNA sequences implies it being the chromosome of biological and evolutionary significance^25^,^26^.

The product of the *UQCR* gene is the ubiquinol-cytochrome c reductase complex, also called mitochondrial complex III. It functions to form a part of the mitochondrial respiratory chain. It may also act as a binding factor for the iron-sulfur protein. Mitochondrial Complex III is composed of one mitochondrial-encoded subunit (MT-CYB) and ten nuclear-encoded subunits. The complex is located within the mitochondrial inner membrane and plays an important role in biochemical synthesis of ATP. It functions to catalyze electrons to transfer from succinate and nicotinamide adenine dinucleotide linked dehydrogenases to mitochondrially encoded cytochrome b. It also functions to utilize the energy to translocate protons across the membrane^27^. Deficiency of isolated complex III has been detected in patients of neuromuscular and nonneuromuscular disorders in both children and adults^28^.

*TCF3* (Transcription factor 3) gene encodes two basic helix-loop-helix(HLH) transcription factors E12 (synonyms–Immunoglobulin Transcription Factor-1 (ITF1)) and E47 (Transcription Factor-3 (TCF-3))^29^. These two factors are believed to function in the regulation of cell growth, differentiation of muscle cells, and commitment. The mechanism is that they act as dimerisation partners for lineage-specific HLH proteins such as the myogenic factors myoblast determination (Myod) and myogenin (Myog)^30^.

The processes of myogenic specification and differentiation are regulated by a family of myogenic basic HLH transcription factors that include Myod, Myog, myogenic factor 5 (Myf5), and myogenic factor 6 (Myf6) in the human^31^. These heterodimers yield a sophisticated pathway (Supplemental Fig. 1) that leads to the expression of critical genes that are specific to muscle. They also promote precursor cells (myoblasts) to develop into multinucleated and differentiated muscle cells (myotubes)^32^.

The *MBD3* (methyl-binding 3) gene encoded protein is a subunit of the NuRD, a multi-subunit complex comprised of Hdac1 (histone deacetylase1), Hdac2, Rbbp7 and Rbbp4 containing nucleosome remodeling and histone deacetylase activities^33^. The class I histone deacetylase Hdac1 is directed associated with MyoD. It is capable of deacetylating MyoD and blocking the function of the latter from initiating the myogenic program under differentiation conditions^34^. In differentiating muscle cells, the reduced level of *HDAC1* is associated with increased level of MyoD acetylation, which stimulates transcription^35^.

Despite their potential relevance to lean mass development, it is unlikely that all the three identified genes are causal. This is because these genes are identified through statistical association instead of biological validation. Statistical association is affected by LD structure, and is usually called indirect gene mapping. These three genes have overlapping test regions, and they present strong levels of LD.

To further explore their potentially causal role, we used a recently developed method PrediXcan to analyze the three identified genes^36^. In brief, the PrediXcan method imputes the unobserved gene expression levels of the GWAS samples, and examines the association between imputed gene expressions and the trait. Unfortunately, none of the three identified genes is significant by the PrediXcan method (p > 0.05). We recognized that the PrediXcan method relies on the eQTL effect of SNPs to the analyzed gene. To evaluate whether the eQTL effect is present, we searched the GTEx datasets through Haploreg^37^, and found that the eQTL effects are very limited for analyzed SNPs. For example, of the 34 SNPs used to calculate the *UQCR* gene signal, none has eQTL activity to its expression. The determination of causal genes are still awaiting further functional studies.

In summary, we identified the 19p13.3 region containing *UQCR, TCF3* and *MBD3* genes, which were significantly associated with LMI in the Caucasian population. These results, together with the known functions of the three genes related to LBM, support that the 19p13.3 region containing *UQCR TCF3* and *MBD3* genes is a novel locus underlying human LBM.

## Additional Information

**How to cite this article**: Ran, S. *et al*. Gene-based genome-wide association study identified 19p13.3 for lean body mass. *Sci. Rep.* **7**, 45025; doi: 10.1038/srep45025 (2017).

**Publisher's note:** Springer Nature remains neutral with regard to jurisdictional claims in published maps and institutional affiliations.

## Figures and Tables

**Table 1:** Basic characteristics of the study subjects.

	*Discovery Sample (Caucasian*)		*Replication Sample (Caucasian*)	
	*Male*	*Female*	*Male*	*Female*
No. of subjects	501	499	556	1727
Age	50.65 (18.60)	50.00 (17.90)	50.71 (16.05)	51.59 (12.92)
Height (m)	1.77 (0.07)	1.64 (0.06)	1.71 (0.07)	1.62 (0.03)
Weight (kg)	89.20 (14.90)	71.39 (16.10)	87.08 (16.70)	71.46 (16.00)
Fat body mass (kg)	23.46 (8.88)	26.92 (10.33)	20.67 (9.30)	20.92 (13.20)
Lean body mass (kg)	63.67 (8.22)	43.49 (6.69)	66.60 (9.59)	46.85 (7.04)
LMI	2.01 (0.22)	1.62 (0.23)	2.15 (1.04)	1.75 (1.31)

**Table 2:** Genes identified for LMI by gene-based association test.

Gene	Chr	Start	Stop	No. SNPs	Discovery		Replication		Combined
					z	p	z	p	p
*UQCR*	19	1548170	1556431	30	2.36	6.10 × 10^−5^	0.30	2.21 × 10^−3^	**2.26** × **10**^**−6**^
*TCF3*	19	1560294	1601277	51	1.84	1.65 × 10^−4^	0.41	1.84 × 10^−3^	4.86 × 10^−6^
*MBD3*	19	1527677	1543652	47	1.82	1.10 × 10^−4^	0.18	6.95 × 10^−3^	1.15 × 10^−5^

Notes: Z-score was the average z-scores over all included SNPs. z-score at each SNP was referred to the increasing-allele in the discovery sample whose z-score was positive by definition. Genome-wide significance (GWS) level significant result was marked in bold.
